# Elbow Disorders in an Outpatient Clinic: An Epidemiological Study

**DOI:** 10.7759/cureus.85029

**Published:** 2025-05-29

**Authors:** Leonardo Zanesco, Rafael Uthant, Caique Arai, Jorge Assunção, Caio Checchia, Rodrigo A Beraldo, Eduardo A Malavolta

**Affiliations:** 1 Orthopedics, Hospital das Clinicas da Faculdade de Medicina da Universidade de Sao Paulo, Sao Paulo, BRA; 2 Orthopedics, Hospital do Coraçao - HCor, Sao Paulo, BRA; 3 Medicine, Faculdade de Medicina da Universidade de Sao Paulo, Sao Paulo, BRA; 4 Orthopedics, Diagnostico das Americas S.A. Hospital 9 de Julho, Sao Paulo, BRA; 5 Orthopedics, Hospital Sírio-Libanês, Sao Paulo, BRA; 6 Orthopedics, Instituto Jundiaiense de Ortopedia e Traumatologia, Jundiai, BRA

**Keywords:** elbow injuries, elbow joint pain, epidemiologic studies, outpatient clinic, public health policy

## Abstract

Background: Elbow disorders are a common cause of pain and functional impairment in the adult population, yet there is a lack of comprehensive epidemiological studies describing their distribution. This study aims to fill this gap by analyzing the epidemiology of elbow complaints in a specialized orthopedic clinic.

Objective: To assess the prevalence, age distribution, and sex-related differences of the most common elbow disorders in an outpatient orthopedic setting.

Methods: A single-center cross-sectional study of medical records from patients treated between 2015 and 2024. Adults of both sexes presenting with elbow pain or dysfunction confirmed by imaging were included. Acute fractures and dislocations were excluded. Diagnoses were classified into major categories, including but not limited to lateral and medial epicondylitis, olecranon bursitis, cubital tunnel syndrome, osteoarthritis, tendinopathies, stiffness, and instability. Data were analyzed descriptively, with age categorized into 10-year intervals.

Results: A total of 847 patients were analyzed, with a mean age of 48.4 ± 14.5 years, and 55.2% were male. The most prevalent diagnosis was lateral epicondylitis (53.2%), followed by medial epicondylitis (16.2%) and olecranon bursitis (5.8%). The highest incidence occurred in the 50-59 age group. Based on the data presented, the greatest discrepancy between genders occurred in olecranon bursitis and cubital tunnel syndrome, with male predominance of 77.6% and 58.3%, respectively, but overall both men and women were more affected by lateral epicondylitis.

Conclusion: In our investigation, lateral epicondylitis (53.2%), medial epicondylitis (16.2%), olecranon bursitis (5.8%), triceps tendinopathy (5.0%), and elbow stiffness (4.4%) were identified as the five most frequent diagnoses with an overall slight male prevalence.

## Introduction

Elbow disorders represent a substantial cause of pain and impairment within the general population. Elbow-related discomfort, a common condition affecting 1-3% of the adult population, varies in occurrence depending on the studied demographic and diagnostic criteria used [1]. In contrast to shoulder disorders, which have a well-established prevalence with extensive literature detailing their distribution across various groups, including the general population, athletes, and children, elbow disorders lack such comprehensive epidemiological data [2-4].

In adults, the most frequent causes of elbow pain are overuse-related injuries such as lateral epicondylitis (tennis elbow), medial epicondylitis (golfer's elbow) and degenerative conditions like primary elbow osteoarthritis, according to a Japanese cohort [5]​. Other conditions include ulnar nerve entrapment and triceps tendinopathy, often related to occupational or athletic activities involving repetitive elbow flexion and extension [1].

Musculoskeletal disorders of the upper extremities significantly impact quality of life and economic outcomes. These conditions account for a substantial proportion of lost work time and increased healthcare burden [6]​. The financial implications are considerable. For instance, lateral epicondylitis alone results in significant direct medical costs and diminished productivity [7]​. Furthermore, patients with chronic elbow disorders frequently experience emotional and psychological distress due to persistent pain and limitations in everyday activities [8]​. Such disorders also contribute to disability and markedly reduce upper extremity-related quality of life [8].

Despite the significance of elbow disorders, there is a scarcity of data regarding their overall epidemiological distribution. To our knowledge, no comprehensive study has elucidated the prevalence and demographic distribution of elbow disorders in the general population, being limited only to the setting of occupational hazards or athletic populations. This study aims to address this gap by delineating the prevalence of various elbow disorders and their distribution by age and sex in an outpatient orthopedic clinic. Elucidating the epidemiological profile of these disorders can better inform clinicians and policymakers regarding diagnostic strategies, resource allocation, and preventive interventions.

## Materials and methods

Study design

This is a cross-sectional study of medical records from patients treated by senior author Eduardo A. Malavolta, an orthopedic surgeon with 18 years of experience in elbow surgery, in a single center. The study was approved by the institutional review board (approval number 80728924.8.0000.0068). Patients of all ages who presented between 2015 and 2024 were included. Patients with acute fractures and dislocations, lack of information for a precise diagnosis, or with symptoms unrelated to the elbow were excluded. Henceforth, all mentions of fractures and dislocations will pertain only to resulting chronic disorders.

Data collection

The database was constructed using FileMaker (FileMaker Incorporated, Santa Clara, USA), generating an Excel (Microsoft Corporation, Redmond, USA) spreadsheet containing patient demographics and diagnosis information. Age was recorded in full years at the time of the first consultation and categorized into 10-year intervals. Diagnosis was classified as follows: lateral epicondylitis, medial epicondylitis, olecranon bursitis, cubital tunnel syndrome, stiffness, instability, osteoarthritis, distal biceps tendinopathy, triceps tendinopathy, elbow dislocation, and myofascial syndrome. When diagnosis prevalence was lower than 0.5%, it was categorized as “other”. For cases with multiple complaints, only the most clinically significant diagnosis was selected. Age and sex distribution were determined for the most frequent diagnoses.

Statistical analysis

Data were presented descriptively. Continuous variables were expressed as means and standard deviations, while categorical variables were presented as total numbers and percentages. Data analysis was performed using Stata 18.0 BE (StataCorp LLC, College Station, USA).

## Results

We analyzed medical records of 962 patients presenting with elbow complaints. Following the exclusion of patients with insufficient data for a precise diagnosis, the final study population comprised 847 participants. The mean age was 48.4 ± 14.5 years, with 55.2% being male. The youngest patient was two years old and the oldest was 97. Overall distribution of diagnosed elbow diseases is described in Table 1.

**Table 1:** Absolute and percentage diagnoses affecting the elbow

Diagnosis	n	%
Lateral Epicondylitis	451	53.2
Medial Epicondylitis	137	16.2
Olecranon Bursitis	49	5.8
Triceps Tendinopathy	43	5.0
Stiffness	37	4.4
Cubital Tunnel Syndrome	37	4.4
Instability	30	3.5
Osteoarthritis	24	2.8
Distal Biceps Tendinopathy	14	1.6
Myofascial Syndrome	8	1.0
Others	17	2.1
Total	847	100

The most frequent diagnosis were lateral epicondylitis, comprising 53.2% of cases. This condition exhibited a slight male predilection (54.6% vs. 46.8% in females), with the highest incidence observed in individuals aged 50-59 years (32.3%), irrespective of gender. Medial epicondylitis emerged as the second most common diagnosis, accounting for 16.2% of cases, also displaying a male predominance (62.0%), with the highest frequency observed in the 50-59 age range (38.6%), although cases were documented as early as the second decade of life. 

**Figure 1:**
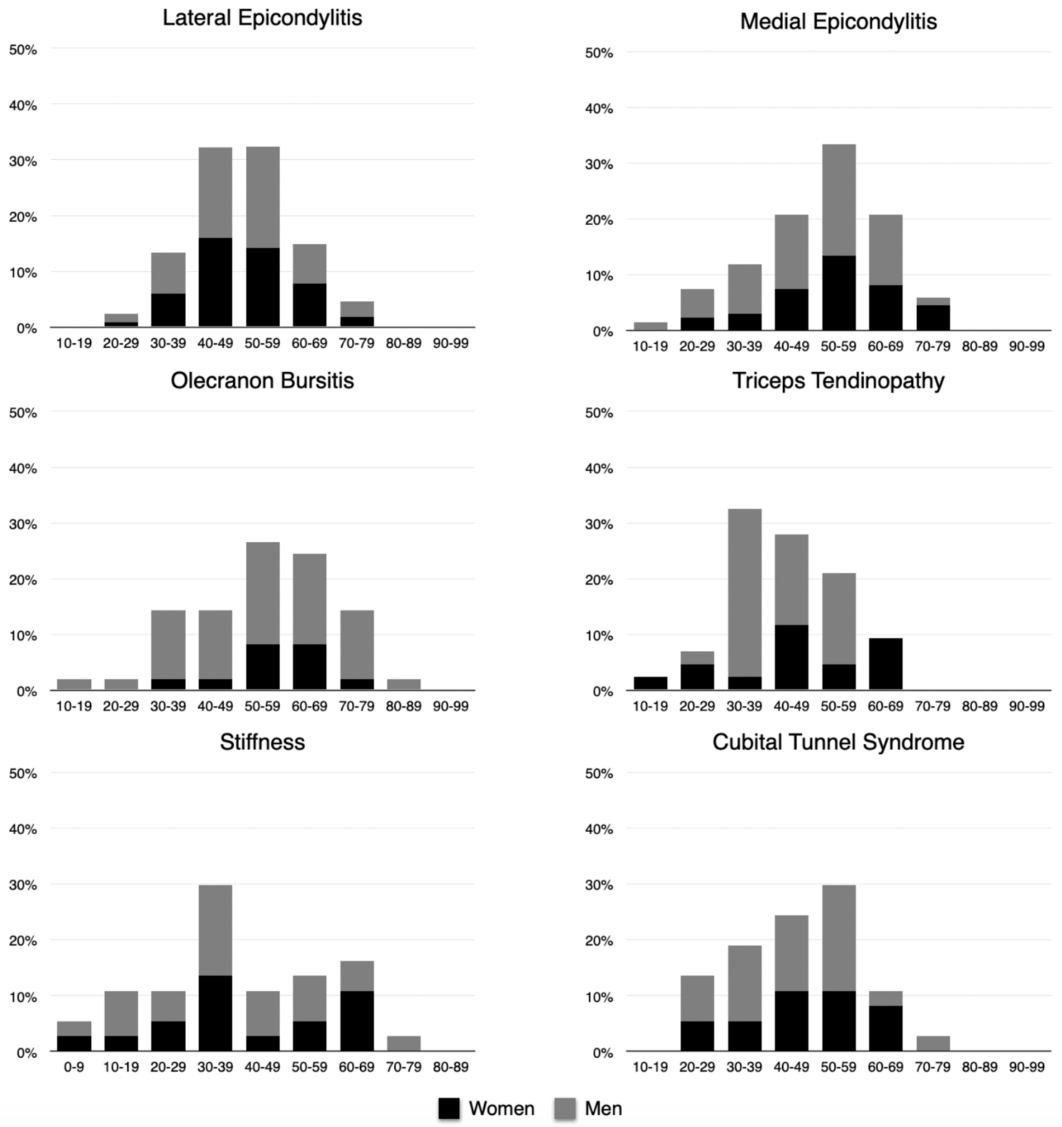
Percentage distribution of primary diagnoses by decade of life and gender

In the study, olecranon bursitis accounted for 5.8% of cases, with a notable male predominance (77.6%). The age distribution revealed that male patients were most commonly affected in the 50-59 year bracket (23.6%), whereas females showed peak incidence between 60 and 69 years (36.3%). Additionally, triceps tendinopathy (5.0%), elbow stiffness (4.4%), and cubital tunnel syndrome (4.4%) all demonstrated higher prevalence among males. The age and gender distribution patterns for these frequently encountered diagnoses are visually represented in Figure 1.

## Discussion

Lateral and medial epicondylitis constituted 69.4% of total elbow complaints in the outpatient setting. The prevalence of elbow injuries appears to be highest among individuals aged 30 to 59 years, with a slight predominance observed in the male population (55.2%). Stiffness and instability accounted for nearly 10% of elbow consultations, typically presenting as a combination of pain and functional limitation. These cases are often complex and frequently result from suboptimal management of an acute elbow injury [9,10].

Elbow disorders impose a significant health and economic burden. Lateral epicondylitis, commonly known as tennis elbow, has been reported to affect 1-3% of adults annually [11]​. This condition frequently leads to functional impairment and may necessitate prolonged treatment, including physical therapy and, in refractory cases, surgical intervention [7]​. Studies indicate that work-related physical risk factors, particularly highly repetitive movements and forceful exertions, significantly contribute to the etiology of epicondylitis [12]​. Furthermore, a systematic review identified a strong association between work-related physical demands and elbow disorders, emphasizing the role of occupational exposure in disease pathogenesis​.

Beyond the physical implications, elbow disorders also present psychosocial and financial challenges. Individuals with persistent symptoms frequently experience reduced work productivity, absenteeism, and job loss [13]​. A population-based study revealed that 16% of individuals with lateral epicondylitis missed work, with an average sick leave duration of 9.3 weeks over the year [14]​. In Tunisia, musculoskeletal disorders of the elbow accounted for 8.35% of all recognized occupational diseases, with a significant prevalence among textile industry workers​ [13]. Given the occupational burden, there is an urgent need for preventive strategies, workplace ergonomics improvements, and early diagnosis to mitigate long-term disability and healthcare costs.

The Metropolitan Region of Sao Paulo, with an estimated population of approximately 19.5 million inhabitants in 2022, demonstrates a slight female predominance (52.1% female vs. 47.9% male) and an age distribution primarily composed of adults within the economically active range [15]. According to the 2022 census, individuals aged between 30 and 59 years account for over 55% of the population, with the highest representation in the 30-39 (16%) and 40-49 (15.4%) age groups [15]. These demographic characteristics are consistent with those observed in our sample, in which the majority of patients presenting with elbow complaints were between 30 and 59 years of age, with a mean age of 48.4 years. The similarity in age distribution between the general regional population and our study sample suggests that our findings are locally applicable, particularly in informing occupational health policies and preventive strategies targeted at this predominant age group.

This study exhibits several limitations that warrant consideration. Firstly, the reliance on retrospective medical records and inclusion of only patients seeking medical attention may introduce selection bias. The decision to exclude fractures and dislocations due to their infrequency in the outpatient setting may underestimate the prevalence of elbow injuries in the population. Furthermore, given that the study was conducted in a specialized orthopedic outpatient setting its findings may not be fully generalizable to the general population, particularly individuals managed in primary care, public health systems, or occupational health settings.

Notwithstanding these limitations, our findings contribute substantial epidemiological data regarding elbow disorders, an area with limited prior research. The results are consistent with previous studies reporting high prevalence rates of lateral and medial epicondylitis, reinforcing their clinical and economic significance [11]​. Furthermore, elucidating the age and sex distribution of these conditions can facilitate the development of preventive strategies and inform government policies aimed at mitigating the burden of work-related musculoskeletal disorders. Subsequent studies should focus on longitudinal analyses, functional outcomes, and intervention effectiveness to further advance knowledge in this field.

## Conclusions

In our investigation, lateral epicondylitis (53.2%), medial epicondylitis (16.2%), olecranon bursitis (5.8%), triceps tendinopathy (5.0%), and elbow stiffness (4.4%) were identified as the five most frequent diagnoses. Lateral epicondylitis exhibited a higher frequency in males (54.6%) compared to females (46.8%), with peak occurrence observed in the 50-59 age cohort for both sexes. These findings highlight the importance of targeted preventive strategies, focused on the most common disorders, to reduce the clinical and economic burden of such conditions.

## References

[1] Kane SF, Lynch JH, Taylor JC (2014). Evaluation of elbow pain in adults. Am Fam Physician.

[2] Malavolta EA, Gracitelli ME, Assunção JH, Pinto GM, da Silveira AZ, Ferreira AA Neto (2017). Shoulder disorders in an outpatient clinic: an epidemiological study. Acta Ortop Bras.

[3] Asker M, Brooke HL, Waldén M, Tranaeus U, Johansson F, Skillgate E, Holm LW (2018). Risk factors for, and prevention of, shoulder injuries in overhead sports: a systematic review with best-evidence synthesis. Br J Sports Med.

[4] Dashe J, Roocroft JH, Bastrom TP, Edmonds EW (2013). Spectrum of shoulder injuries in skeletally immature patients. Orthop Clin North Am.

[5] Nakayama K, Kato H, Ikegami S (2022). Prevalence and associated factors of primary elbow osteoarthritis in the Japanese general elderly population: a Japanese cohort survey randomly sampled from a basic resident registry. J Shoulder Elbow Surg.

[6] Walker-Bone K, Palmer KT, Reading I, Coggon D, Cooper C (2004). Prevalence and impact of musculoskeletal disorders of the upper limb in the general population. Arthritis Rheum.

[7] Sanders TL, Maradit Kremers H, Bryan AJ, Ransom JE, Morrey BF (2016). Health care utilization and direct medical costs of tennis elbow: a population-based study. Sports Health.

[8] Moon DK, Park YJ, Song SY (2018). Common upper extremity disorders and function affect upper extremity-related quality of life: a community-based sample from rural areas. Yonsei Med J.

[9] Guglielmetti CL, Gracitelli ME, Assunção JH (2020). Randomized trial for the treatment of post-traumatic elbow stiffness: surgical release vs. rehabilitation. J Shoulder Elbow Surg.

[10] Morrey BF (1996). Acute and chronic instability of the elbow. J Am Acad Orthop Surg.

[11] Sanders TL Jr, Maradit Kremers H, Bryan AJ, Ransom JE, Smith J, Morrey BF (2015). The epidemiology and health care burden of tennis elbow: a population-based study. Am J Sports Med.

[12] Chiarotto A, Gerger H, van Rijn RM (2023). Physical and psychosocial work-related exposures and the occurrence of disorders of the elbow: a systematic review. Appl Ergon.

[13] Khouja N, Hsinet J, Abdennadher K (2024). Occupational elbow musculoskeletal disorders in Tunisia: epidemiology and socio-professional consequences. Tunis Med.

[14] Struijs PA, Korthals-de Bos IB, van Tulder MW, van Dijk CN, Bouter LM, Assendelft WJ (2006). Cost effectiveness of brace, physiotherapy, or both for treatment of tennis elbow. Br J Sports Med.

[15] (2025). Population. https://citypopulation.de/en/brazil/metro/CU3550308__s%C3%A3o_paulo/.

